# Immersion Experiences in a Tablet-Based Markerless Augmented Reality Working Memory Game: Randomized Controlled Trial and User Experience Study

**DOI:** 10.2196/27036

**Published:** 2021-10-12

**Authors:** Bo Zhang, Nigel Robb

**Affiliations:** 1 Department of Education Information Technology Faculty of Education East China Normal University Shanghai China; 2 Research Faculty of Media and Communication Hokkaido University Sapporo Japan

**Keywords:** augmented reality, markerless augmented reality, immersion experience, cognitive training games, working memory, markerless augmented reality n-back game

## Abstract

**Background:**

In recent years, augmented reality (AR), especially markerless augmented reality (MAR), has been used more prevalently to create training games in an attempt to improve humans' cognitive functions. This has been driven by studies claiming that MAR provides users with more immersive experiences that are situated in the real world. Currently, no studies have scientifically investigated the immersion experience of users in a MAR cognitive training game. Moreover, there is an observed lack of instruments on measuring immersion in MAR cognitive training games.

**Objective:**

This study, using two existing immersion questionnaires, investigates students’ immersion experiences in a novel MAR n-back game.

**Methods:**

The n-back task is a continuous performance task that taps working memory (WM) capacity. We compared two versions of n-back training. One was presented in a traditional 2D format, while the second version used MAR. There were 2 experiments conducted in this study that coordinated with 2 types of immersion questionnaires: the modified Immersive Experiences Questionnaire (IEQ) and the Augmented Reality Immersion (ARI) questionnaire. Two groups of students from two universities in China joined the study, with 60 participants for the first experiment (a randomized controlled experiment) and 51 participants for the second.

**Results:**

Both groups of students experienced immersion in the MAR n-back game. However, the MAR n-back training group did not experience stronger immersion than the traditional (2D) n-back control group in the first experiment. The results of the second experiment showed that males felt deeply involved with the AR environment, which resulted in obtaining higher levels of immersion than females in the MAR n-back game.

**Conclusions:**

Both groups of students experienced immersion in the MAR n-back game. Moreover, both the modified IEQ and ARI have the potential to be used as instruments to measure immersion in MAR game settings.

**Trial Registration:**

UMIN Clinical Trials Registry UMIN000045314; https://upload.umin.ac.jp/cgi-open-bin/ctr_e/ctr_view.cgi?recptno=R000051725

## Introduction

In the past decade, studies have reported signiﬁcant improvements in cognitive functions through game-based cognitive training in different groups [1-3]. In recent years, research shows that the application of augmented reality (AR) technology in games is related to the user’s cognitive functionality, as it has a positive impact on mental processes and psychological reactions [4,5]. Many studies also started to explore the affordances of using AR technology to create cognitive training tasks. There is some evidence that AR-based cognitive training is effective in improving users’ cognitive functions, such as attention and memory [6-8]. Other research shows that AR-based tasks play a significant role in increasing students’ motivation and attention during the learning process [9].

By overlapping computer-generated 3D graphics onto a real-world environment, AR enables borderless interactions between the digital and physical worlds [10]. AR has the following key features: combination of virtual content and real context, real-time interaction between virtual objects and real contexts, and the registered 3D virtual object [11]. Through mobile devices (eg, smartphones and tablets), AR apps capture the real scene with the camera and then present the digital contents to the real context surrounding the user. This means of interaction creates a new mode of learning and training, which can be easily conducted even by students who have no experience using digital devices [12]. AR apps enable users to have ubiquitous access to surrounding real contextual environments while playing AR games on a mobile interface [13].

According to how AR apps represent virtual graphics in a real context through device camera screens, AR games are divided into 2 types: markerless AR (MAR) games and marker-based AR games [10]. Generally, marker-based AR requires a fiducial or artificial marker placed in a real context to implement AR experiences. These markers, such as printed barcodes and QR codes on cards, generally have specific geometric or color properties, which makes them easy to extract and identify in a video frame. Marker-based AR uses an external marker to calibrate the camera’s pose and then achieves a successful projection of virtual content into the captured real environment [14]. However, this approach has been shown to have many drawbacks, such as virtual content being easily lost in the tracking process if a live camera moves quickly and visual markers have to remain in sight due to a limited range [13,15]. Moreover, AR with fiducial markers is not scalable for outdoor scenes and mobile learning [16].

By contrast, the tracking system (based on a simultaneous localization and mapping technique) of MAR can rely on natural features instead of using external markers to trigger augmentations in the real environment [17]. Through an eligible mobile device, markerless tracking can manipulate any part of a real location as a marker to place virtual objects [18,19]. MAR games are similar to location-aware AR games [20], which are geo-based augmented realities that do not need any special markers for identifying where to place a virtual object in the real environment. In this way, these games can provide more realistic and interactive AR experiences for users, accurately track the real context, and detect locations through smart devices [21]. A MAR app brings a markedly different experience for its users, which enhances interactive involvement with and perception of the real surroundings without physical constrains [5,18,22]. Thus, MAR apps have been argued to provide users with more immersive and interactive experiences, which can be beneficial for promoting enjoyment and engagement in learning and training [21]. As AR technology can enable users to experience a sense of immersion [23,24], MAR game playing may provide users with a stronger sense of immersion experience.

Immersion has been widely discussed in the context of AR games [10,20], especially ones related to cognitive training [6,25]. Immersion, a form of cognitive and emotional absorption, has been used to promote enjoyment and engagement in tasks and learning [26,27]. Immersion is widely considered as a desired outcome of the gaming experience [27]. Performing and training related to immersive digital experiences are assumed to be reliant on the degree of achieved immersion, specifically the degree to which users become cognitively and emotionally engaged with a given digital app [28,29]. These statements indicate that measuring the immersion in the context of an AR game developed for cognitive training is a primary tool to evaluate the effects of AR games. Hence, to discover the potential of MAR games in the field of cognitive training, we need to investigate users’ immersion experiences in MAR games playing. However, there is an observed lack of studies investigating immersion in MAR cognitive training games.

Immersion in the field of digital games has been widely discussed in recent years [20,27,29-31]. The definition of immersion was first proposed in the field of virtual environments. The research by Georgiou and Kyza [20] argues that immersion can be seen as a reliable dimension to objectively access the properties of a virtual environment. On the other hand, Witmer and Singer [32] described the concept of immersion as a “psychological state characterized by perceiving oneself to be enveloped by, included in and interacting with an environment that provides a continuous stream of stimuli and experiences.” In addition, Brooks [28] argued that an immersive experience can not only occur in virtual reality (VR) technologies but also appear when playing simple computer games, such as video games. The deﬁnition of immersion also considers the diﬀerent degrees of cognitive and aﬀective absorption when playing a digital game, as they are closely related to task enjoyment and engagement [20,33]. Moreover, many studies suggest that gender affects the immersion level in digital games [34]. Specifically, men and women report different levels of immersion when interacting with virtual environments [35] and when playing video games [36].

However, the virtual environments described in the definitions were generally created by VR technology. By wearing head-mounted displays, users were immersed in virtual environments, isolating completely from real contexts. While AR games are played using mobile devices, the games can also provide users with immersive experiences by interacting with a continuous stream of digital-based stimuli situated in the real contexts. Hence, users may perceive a different immersive experience when playing AR apps, compared with virtual or other digital environments.

Previously, in the absence of valid immersion measurements, some researchers discussed AR immersive experiments through the evaluation of ﬂow and presence [37,38]. Csikszentmihalyi [39] defined flow as “the state in which individuals are so involved in an activity that nothing else seems to matter.” As for the immersion state, ﬂow can be described as people so absorbed in their activities that irrelevant thoughts and perceptions are filtered out from their minds [27]. The definition of presence is a psychological sense of being in a virtual environment [40]. The degree to which the virtual environment mimics real-world experiences is impacted by the degree of participants’ presence in the virtual environment [32].

Some studies argued that the sense of ﬂow and presence could barely be created and maintained in AR games [38], as potential external distractions exist in the context of an AR system. For instance, many factors such as temperature, light and noise can be easily controlled in the virtual environment, while these parameters are hardly controlled by designers of AR apps in a real context [41]. These potential and uncontrolled external distractions may prevent users from giving their attention and thus disrupt their immersive experiences in AR games [38,41].

In addition, Benyon [42] stated that immersion in AR experiences is different from feelings of presence, in terms of being in a virtual space. Yet, immersion in AR experiences is the feeling of involvement in a blended space of real and digital elements. Moreover, a good AR game should be able to immerse its users into playing the game and decrease their focus on external distractions situated in real contexts. Hence, the evaluation of immersion provides a valuable option to describe the user experiences in the context of AR (or MAR) games.

There are several previously developed validated instruments for evaluating immersion in digital games [20,27,29,30]. Although these existing instruments are validated in some games, they may be invalidated in other types of games (eg, AR games) when measuring the immersion experience. As described above, we can see MAR games are technically and functionally different from other digital software (eg, 2D games), as they can present 3D elements and software assets in real environments without external markers.

It is uncertain which existing valid immersion questionnaire is valid for measuring user immersion in emerging MAR apps. As a potential benefit of MAR technology in developing cognitive training games, identifying valid instruments for evaluating immersion in MAR games is significant for the researchers and developers in this field. Therefore, this study aims to explore students’ immersion experiences in a novel MAR cognitive training task by using 2 existing immersion instruments for digital games.

## Methods

### Development of a MAR n-Back Task

**Figure 1:**
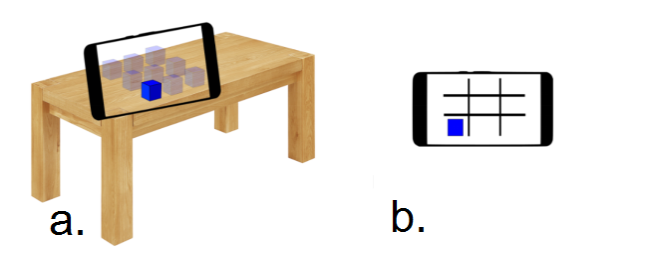
Examples of (a) markerless augmented reality n-back game and (b) traditional (2D) n-back game.

An n-back task is used extensively in the literature as a working memory (WM) training task [43,44]. Participants in an n-back task are presented with a series of stimuli. They are required to retain some aspects of each stimulus in WM (eg, the location of the stimulus in a grid). During the n-back task, the trainee is instructed to respond whenever a current stimulus is presented on-screen that matches the one presented n positions back in the sequence [43]. In this study, a MAR n-back game and a 2D n-back game were developed (Figure 1) based on the same principles of the n-back task described in previous studies [45-47]. The MAR n-back game was developed using a combination of the Unity game engine and Apple’s ARKit database, based on the MAR game framework developed by Chen et al [14] that could achieve real-time 3D context reconstruction by using mobile devices. The main differences between the 2 versions of the game were how the player perceived the location of the stimuli: whether these stimuli were more stimulating graphics and whether these stimuli were perceived as being located on the screen of the device (non-MAR) or as being in real-world surroundings (MAR).

**Figure 2:**
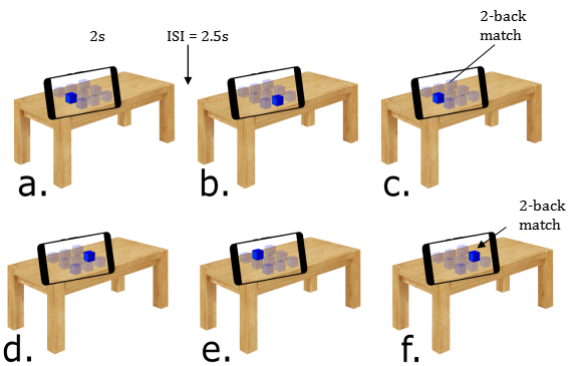
Six successive trials from the augmented reality version of the n-back game.

Figure 2 shows 6 successive trials from the MAR n-back game. Each trial consisted of a stimulus that was presented for 2 seconds (s), followed by an interstimulus interval of 2.5 s and the next stimulus [43]. In this example, with n (n=1, 2, or 3) set to 2 (2-back), the correct responses would be to indicate a match on trial (c) because the location matches the trial’s previous 2 steps (a), and to indicate a match on trial (f) because the location matches the trial’s previous 2 steps (d).

**Figure 3:**
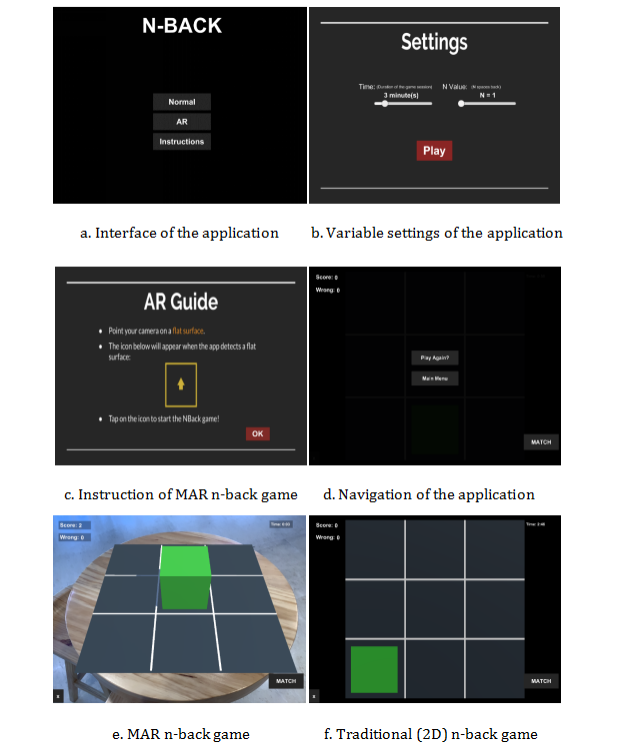
App settings of both versions of the n-back game. MAR: markerless augmented reality.

**Figure 4:**
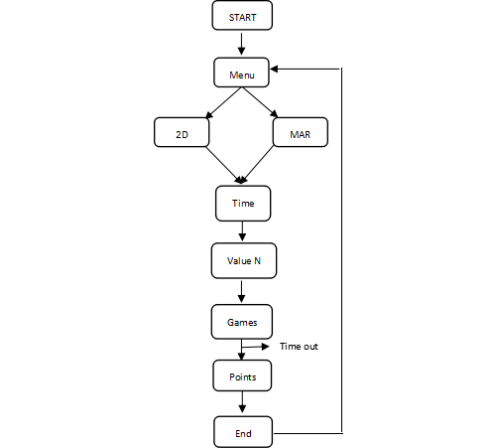
Flowchart of a full gaming session with both versions of the n-back game. MAR: markerless augmented reality.

Hence, by playing the MAR n-back game, participants can interact with stimulus within the real context via smart devices. We assume that MAR n-back games could provide users with more immersive and interactive experiences, thereby being potentially beneficial for promoting enjoyment and engagement in cognitive training. As AR technology has been increasingly applied to various domains, it is urgent to investigate the essential theories and practices related to the key aspects of AR experiences, such as immersive features. In this study, both versions of the n-back games were created with 3 load levels (1-back, 2-back, and 3-back). Figure 3 illustrates the app settings of both versions of the n-back game, while Figure 4 displays the flowchart of a full gaming session (spanning 3 minutes). Hence, the motivation of n-back game training is based on the participants responding to the correct targets as quickly and accurately as possible, while the difficulty increases by raising the value of n. Through this way, participants were motivated to take the n-back game training seriously and perform effectively, as their scores were cumulatively displayed against other players.

### Immersion Measurements

#### Background

In this study, two existing validated immersion instruments: the Immersive Experiences Questionnaire (IEQ) and the Augmented Reality Immersion (ARI) questionnaire have been selected as the measurements with some reasons.

In 2004, Brown and Cairn [30] created a famous model of immersion with the 3 sequential levels of engagement, engrossment, and total immersion to describe the degrees of involvement with digital games. The level of engagement includes access and investment, which means that the player needs to invest time and effort in learning how to play the game and get familiar with the game’s controls. From the engagement level, participants may be able to become more interested in and further involved with the game and then move into the engrossment level. During this level, gamers’ attention spans and emotions were directly affected by the game and game controls became invisible because gamers became less aware of their surroundings [27]. Total immersion occurred when gamers reached a sense of presence, became lost in the game’s world, and achieved a sense of feeling that the game was all that mattered.

On the basis of immersion model [30], ﬂow [39], cognitive absorption [33] and presence [32], the IEQ was developed for measuring immersion in digital games [27]. In particular, the validity of the IEQ was proven through experiments with 2 different types of games: a video (2D) and a VR game. As virtual environments, although AR differs from VR in displaying digital content, both technologies are claimed to represent effective immersive technology [48]. Moreover, the focus of the IEQ covers 5 factors: cognitive involvement, emotional involvement, real-world dissociation, challenge, and control [27]. These factors are more related to the purpose of measuring immersion in a cognitive training game (eg, n-back game). Hence, using the IEQ, this study sets a similar experiment to explore differences in students’ immersion experiences with 2 different types of games: a video (2D) game and a MAR game.

The ARI questionnaire, based on the immersion theory of Brown and Cairns [30], was developed particularly for the purpose of measuring immersion in location-aware AR settings, which, as explained earlier, is similar to MAR games. It has also been used for measuring immersion in other types of AR apps used for training [31]. Georgiou and Kyza [20] emphasize that immersion in AR apps is not about getting disengaged from the real world but shifting attention toward AR games, which in turn results in a decreased focus on any potential distractors. Hence, to investigate students’ immersion experiences in a MAR game, the ARI questionnaire is necessary for this study.

#### Immersive Experiences Questionnaire

The IEQ includes 33 items; 32 questions (Q1-Q32) were all designed on 5-point scales (1 = strongly disagree and 5 = strongly agree). One question used a 10-point scale, asking participants to indicate how immersed the felt overall (1 = not at all and 10 = very much).

However, as discussed in the previous section, users perceive a feeling of being surrounded by a blended world of real and digital elements in AR games, unlike virtual environments in which users can totally immerse themselves [36].

Therefore, to make the IEQ suitable for assessing immersion in AR game settings, some questions were omitted:

- Q19: It was as if I could interact with the world of the game as if I was in the real world.
- Q20: Interacting with the world of the game did not feel as real to me as it would be in the real world.
- Q23: I felt detached from the outside world.
- Q30: I still felt as if I was in the real world while playing.

Moreover, certain potential external distractions, such as weather and noises, exist in the context of an AR system. Therefore, the Q21 “I was unaware of what was happening around me” should also be removed from the IEQ [20]. Hence, a modified IEQ, with 27 5-point scale questions (omitting questions Q19, Q20, Q21, Q23, Q30) and one 10-point scale question, was used to measure participant immersion experiences in this study.

#### ARI Questionnaire

The ARI questionnaire is a 21-item, 7-point Likert-type instrument ranging from totally disagree (1) to totally agree (7) [20]. The ARI questionnaire has a 3-level construct (with each construct broken down further into 2 subcategories) according to the following: engagement (interest and usability), engrossment (emotional investment and focus of attention), and total immersion (presence and flow). These factors measure the immersion level of students while using AR apps.

### First Experiment

#### Participants

Recruitment of participants began in October 2019. A total of 60 participants took part in the first experiment (average age 18.97 [SD 1.09] years; 42 females). They were a group of undergraduate students from a university in China. No eligibility criteria were specified. Written informed consent forms were collected from these students before they began the WM training. Participants were informed that they could drop out of the study at any time. Prior to beginning the study, ethics approval was obtained from the ethics committee at East China Normal University (HR 055-2019). The trial was registered at the University Hospital Medical Information Network Clinical Trials Registry [UMIN000045314]. All participants were paid US $15.

#### Procedure

**Figure 5:**
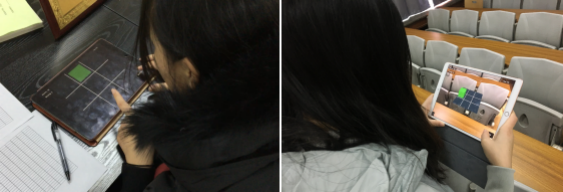
Visual depictions of the first experiment.

At the beginning of the first experiment, participants were randomly divided into 2 groups, with 30 participants in the control group (who trained on the video n-back game) and 30 in the experimental group (who trained on the MAR n-back game). Participants were not blinded. Random allocation was based on random numbers generated in Microsoft Excel by BZ. During the training, six 10.5-inch iPad Pro tablets (Apple Inc) were used to present the n-back game (see Figure 5). The instructions for the n-back task were demonstrated to the participants prior to the WM training. Two groups of participants then received separate WM training in 2 versions of n-back tasks for 8 sessions (2 sessions a day, with each session lasting 3 minutes) within 4 days. All training sessions were conducted in a laboratory. At this stage, the aim of the training was not to investigate its effects on WM but to focus on comparing the level of immersion between the 2 groups. After all the training sessions were completed, each participant completed a modified IEQ through a link to an online questionnaire using their mobile phones.

### Second Experiment

#### Participants

A total of 51 participants took part in the second experiment (average age 21.22 [SD 1.69] years; 30 females). Participants, studying in the major of Educational Technology, were recruited from another university in China. To complete the assignments on the evaluation of an innovative software, these students need to test an innovative app. All participants were fully debriefed at the end of the experiment. Written informed consent forms were also collected from these students before they completed the survey.

#### Procedures and Materials

**Figure 6:**
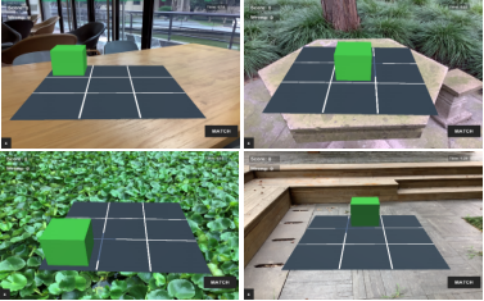
Visuals demonstrating the second experiment.

In this experiment, 51 participants practiced in a MAR n-back game for once a day, 3 minutes each time, using 6 iPad tablets for 1 week. Participants were allowed to take the tablets with them outside of the classes and could play the MAR n-back game at any location within the university’s campus. The instructions for the WM training with MAR n-back game were explained to the participants and several practice trials were conducted before the initiation of the study. Some visuals illustrating the practice process are presented in Figure 6.

After 1 week of playing, the ARI questionnaire was issued to students to complete through an online link using mobile phones, and data were collected. The practice process was given separately to each participant and the data collection took place over a 1-week period. As the ARI questionnaire is particularly developed to measure immersion in AR settings, the second experiment aims to further investigate students’ immersion experiences in a MAR game based on the study of Georgiou and Kyza (2016).

## Results

### First Experiment

Following the example of Jennett et al [27], we first examined correlations between the 2 immersion measures of 27 5-point scale questions and one 10-point scale question in the modified IEQ. Taking both groups (control and experimental groups) together, the 2 immersion measures were positively, significantly correlated (Pearson *r*=0.466, *P*<.001). This finding appears reliable, as variables such as participant age, gender, and education level were all found to have nonsignificant correlations with both immersion measures. Taking the groups individually, the correlation was stronger in the MAR n-back group (Pearson *r*=0.518, *P*=.003) than the 2D n-back group (Pearson *r*=0.362, *P*=.049). Based on a total 28 items of the modified IEQ, while the MAR n-back group had a higher mean value on all immersion measures (see Table 1) than the 2D n-back group, the differences between the 2 groups were not significant on either measure.

**Table 1:** Mean and standard deviation for the measures of immersion in the modified Immersive Experiences Questionnaire.

Characteristic	5-point scale (27 items)		10-point scale (1 item)	
	MAR^a^ (n=30)	2D^b^ (n=30)	MAR (n=30)	2D (n=30)
Mean	3.584	3.421	7.9	7.433
Standard deviation	0.619	0.516	1.668	1.501

^a^MAR: markerless augmented reality n-back game.

^b^2D: n-back game.

### Second Experiment

Cronbach alpha for the ARI scale was 0.783 overall and ranged from 0.711 to 0.836 for the 6 subdimensions (ie, interest, usability, emotional investment, focus of attention, presence and flow), indicating acceptable reliability. The mean values for engagement, engrossment, and total immersion with the subdimensions of interest, usability, emotional investment, focus of attention, presence, and flow (see Table 2) were similar to the research by Georgiou and Kyza [20] using the ARI to investigate immersion in AR experiences but noticeably lower than that found in the research of Salar et al [31].

**Table 2:** Mean values for interest, usability, emotional investment, focus of attention, presence and flow.

Factor	Value, mean (SD)
Interest	5.853 (0.156)
Usability	3.995 (0.961)
Emotional investment	4.580 (1.697)
Focus of attention	4.954 (0.300)
Presence	4.299 (0.388)
Flow	4.848 (0.150)

**Figure 7:**
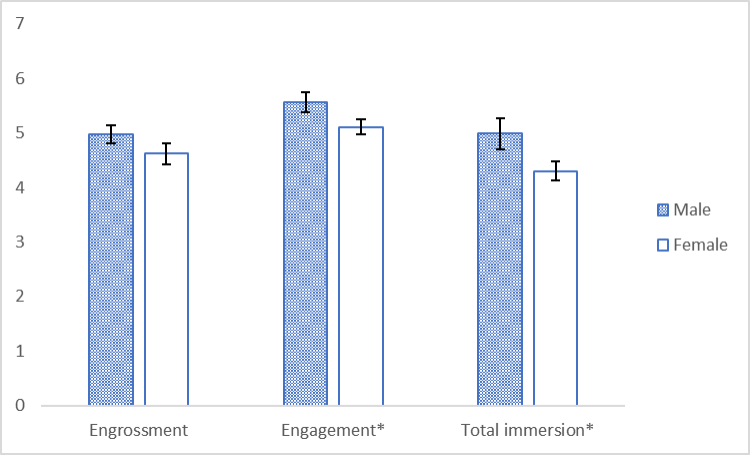
Mean values for engrossment, engagement, and total immersion for males and females. Significant differences (*P*<.05) are marked with an asterisk (*).

However, males reported higher mean values than females on all 3 constructs of the immersion model (see Figure 7). These differences were significant in the case of engagement (Student *t* test *P*=.048, *P*<.05) and total immersion (Student *t* test *P*=.033, *P*<.05; see Table 3 and Figure 7).

**Table 3:** Differences between males and females on mean values for engrossment, engagement and total immersion.

Construct	Male	Female	Student *t* test		
	Mean (SD)	Mean (SD)	*t*	*df* ^a^	*P* value
Engagement	5.565 (0.849)	5.108 (0.750)	–2.029	49	.048
Engrossment	4.976 (0.775)	4.622 (1.063)	–1.301	49	.20
Total immersion	4.994 (1.307)	4.303 (0.950)	–2.189	49	.03

^a^*df*: degree of freedom.

## Discussion

### Principal Findings

Recently, many researchers have paid increasing attention to the application of AR games for cognitive development. However, with the lack of validated instruments for measuring users’ immersion in AR apps, the scientific evaluation of emerging AR games is still missing in the literature. Therefore, this study aimed to investigate students’ immersion experiences in a novel MAR n-back game for WM training by using 2 existing immersion questionnaires. As explained earlier, IEQ has the potential to compare users’ immersion with 2 different types of games [27], such as a traditional (2D) n-back and a MAR game; therefore, we used the modified IEQ as the immersion instrument in a randomized controlled experiment. This aims to explore students’ immersion experience with both versions of n-back games and investigate whether students can obtain stronger immersion in the MAR n-back game than the 2D n-back game. However, as the ARI questionnaire has been widely accepted as a valid tool for assessing immersion in the context of AR apps [20], it was used to measure only a single group of students, with the MAR n-back game in the second experiment. The purpose of this experiment was to further explore students’ immersion experiences with the MAR n-back game.

The results show that the correlation between the 2 immersion measures of 27 5-point scale questions and one 10-point scale question of the modified IEQ was positive and significant. This indicates that these 2 different sets of questions were still strongly consistent and connected with each other, which proves the reliability of the modified IEQ. The findings show that while the MAR n-back group had a higher mean value on all immersion measures (see Table 1) than the 2D n-back group, there were no significant differences in the immersion measures between groups on the modified IEQ (27 5-point scale items and one 10-point scale item). The lack of significant differences in immersion may imply that the 2 versions of the n-back games used in this research, although being visually different in many aspects, did not produce obvious different levels of immersion in players. As Figure 1 shows, the traditional (2D) n-back game was played on the screen of a device, and the MAR n-back game was performed in the real environment. Although the MAR n-back game has more simulating graphics, both games have the same motivation rules. Moreover, the participants may be influenced by real-world surroundings, such as sound and light, during the MAR n-back game training in Figure 5 [38,41,42]. It may be that for MAR apps to be more immersive, more is required than simply translating an existing 2D game into an AR version, as we did in this study. On the other hand, participants’ scores on the modified IEQ, in both the 2D and MAR conditions, were similar to the scores in previous research using immersive experiences [27]. This suggests that participants were experiencing immersion in both versions of the n-back training games.

Furthermore, Table 2 shows that the mean values of the second experiment for 6 subdimensions with 51 students all showed relatively higher results compared with the average of the 7-point (average 3.500). We can see participants paid attention to the MAR n-back game and felt it was interesting. These results illustrate that a good level of immersion within participants has been established. However, there is still potential for improvement, as the mean value of the 6 dimensions (see Table 2) are all slightly less than those measured in the study by Salar et al [31]. At this stage, the results of using the ARI questionnaire with participants in the second experiment proved that a MAR n-back game can establish participants’ immersion experiences.

Previous studies argue that immersion is a desired outcome of the gaming experience and can promote participants’ enjoyment and engagement in tasks if their level of involvement increases in the games [27]. Performing and training related to experiences based on immersive technology are assumed to be reliant on the degree of achieved immersion in the games [28,29]. MAR apps bring a different experience for its users, which enhances interactive involvement with the real surroundings without physical constrains [5,18,22]. The findings show that the MAR n-back game (WM training task) can provide users with an immersive and interactive experience, which is beneficial for promoting enjoyment and engagement in training [21]. As the potential affordances of AR-based tasks can improve cognitive functions, such as attention and memory [6-8], this study indicates that the MAR games also have the potential to promote cognition functions (eg, WM in this study), as all the participants experienced immersion during the MAR n-back training.

Unexpectedly, the findings of the second experiment revealed that males gained higher mean values than females on all 3 constructs of the immersion model (see Figure 7), with significantly stronger values for engagement and total immersion (see Table 3 and Figure 7). The immersion theory of Brown and Cairn [30] defines that engagement is the first level of immersion, which indicate that player is willing to invest time and make effort in learning and controlling the game. With the same assignment of manipulating the MAR n-back game, we can see males were willing to make more efforts in controlling the MAR games than females at the first learning stage. However, males and females experienced no significant differences in the engrossment level, which involves more emotional investment on the games. This result may be influenced by the fact that all the students were required instead of self-driven to play the MAR n-back game, hence the emotional input process is short but directly enters the total immersion level. The results also show that male students researched significantly stronger sense of becoming lost in the MAR games (total immersion level) than female students. These results coincide with the finding that male players experienced deeper involvement with the AR environment, which in turn resulted in feeling higher levels of immersion than female players [34].

In conclusion, by conducting 2 experiments by using the modified IEQ and ARI questionnaire, we found that both groups of students were experiencing immersion in the MAR n-back game. As discussed in the Introduction section, MAR games are very different from other digital games as they can present 3D elements and software assets in real environments without external markers. However, there is an observed lack of studies on investigating immersion in MAR games. This study shows that the modified IEQ and ARI questionnaire have the potential to be used as an instrument to measure immersion in MAR settings. Also, as results show that MAR cognitive games can provide users with an immersive and interactive experience, this suggests the affordance of MAR games in improving users’ cognitive functions. However, the MAR n-back game did not produce obvious different levels of immersion in players than the traditional (2D) n-back game in this study, this may imply that the design of the MAR games should have obvious differences from 2D versions or other type of games in order to increase immersion levels. Finally, we found that male players experienced stronger immersion than female players in the MAR n-back game, which further supports that males and females perceive different levels of immersion when interacting with virtual environments [36]. In the end, we believe these findings can contribute to the development of MAR games in the field of cognitive training for AR designers and also for the researchers who are interested in exploring users’ immersion in MAR settings.

### Limitations and Future Study

Although this study has a number of practical implications, there are a number of limitations that must be considered. First, in both experiments, the immersion questionnaire was completed by participants after the practice sessions were completed. Although students finished immersion questionnaires immediately after the game practices, their immersion experiences may not be accurate, as immersion is an instantaneous state that would disappear after the activity is finished. Second, in the second experiment, only a single group of students completed the ARI questionnaire to measure their immersion with the MAR n-back game. Further, a randomized controlled experiment needs to be considered to explore whether the ARI questionnaire can be used to measure immersion within the non-AR games. Third, an experiment with the modified IEQ should be repeated with an app that really differs in an AR version from its 2D version game to see whether still no obvious different levels of immersion in players. It is important to further investigate the affordances of AR technology in building games. Finally, as only one MAR game has been tested using the modified IEQ and ARI questionnaires in this study, more MAR games need to be tested in the future to strengthen the findings of this study.

### Conclusion

In this study, we used two questionnaires, the IEQ and ARI, to investigate immersion in an AR working memory game. In addition, we compared immersion levels between the AR game and a 2D version of the same game. We found that the AR working memory game produced reasonable levels of immersion in players (on both questionnaires), which may contribute to its effectiveness as a cognitive training program. However, there were no significant differences between immersion levels in the AR game and the 2D version (on the IEQ). We also found different levels of immersion experience in men and women on two constructs of the ARI, with men showing significantly higher levels of engagement and total immersion than women in the AR game.

## Appendix

Multimedia Appendix 1 CONSORT-EHEALTH checklist (V 1.6.1).

## Abbreviations

AR: augmented reality
ARI: Augmented Reality Immersion
IEQ: Immersive Experiences Questionnaire
MAR: markerless augmented reality
VR: virtual reality
WM: working memory
